# Interface Engineered Microcellular Magnetic Conductive Polyurethane Nanocomposite Foams for Electromagnetic Interference Shielding

**DOI:** 10.1007/s40820-021-00677-5

**Published:** 2021-07-08

**Authors:** Guolong Sang, Pei Xu, Tong Yan, Vignesh Murugadoss, Nithesh Naik, Yunsheng Ding, Zhanhu Guo

**Affiliations:** 1 Department of Polymer Science and Engineering, School of Chemistry and Chemical Engineering, and Anhui Key Laboratory of Advanced Functional Materials and Devices, Hefei University of Technology, Hefei, 230009 People’s Republic of China; 2 Advanced Materials Division, Engineered Multifunctional Composites (EMC) Nanotech. LLC, Knoxville, TN 37934 USA; 3 Integrated Composites Laboratory (ICL), Department of Chemical and Biomolecular Engineering, University of Tennessee, Knoxville, TN 37996 USA; 4 Department of Mechanical and Manufacturing Engineering, Manipal Institute of Technology, Manipal Academy of Higher Education, Manipal, Karnataka 576104 India

**Keywords:** Polyurethane, Nanocomposites, Microcellular, Electromagnetic interference shielding

## Abstract

Lightweight microcellular polyurethane (TPU)/carbon nanotubes (CNTs)/ nickel-coated CNTs (Ni@CNTs)/polymerizable ionic liquid copolymer (PIL) composite foams are prepared by non-solvent induced phase separation (NIPS). CNTs and Ni@CNTs modified by PIL provide more heterogeneous nucleation sites and inhibit the aggregation and combination of microcellular structure. Compared with TPU/CNTs, the TPU/CNTs/PIL and TPU/CNTs/Ni@CNTs/PIL composite foams with smaller microcellular structures have a high electromagnetic interference shielding effectiveness (EMI SE). The evaporate time regulates the microcellular structure, improves the conductive network of composite foams and reduces the microcellular size, which strengthens the multiple reflections of electromagnetic wave. The TPU/10CNTs/10Ni@CNTs/PIL foam exhibits slightly higher SE values (69.9 dB) compared with TPU/20CNTs/PIL foam (53.3 dB). The highest specific EMI SE of TPU/20CNTs/PIL and TPU/10CNTs/10Ni@CNTs/PIL reaches up to 187.2 and 211.5 dB/(g cm^−3^), respectively. The polarization losses caused by interfacial polarization between TPU substrates and conductive fillers, conduction loss caused by conductive network of fillers and magnetic loss caused by Ni@CNT synergistically attenuate the microwave energy.

**Supplementary Information:**

The online version contains supplementary material available at 10.1007/s40820-021-00677-5.

## Highlights

- Carbon nanotubes/polymerizable ionic liquid copolymer (CNTs/PIL) provides nucleation sites and inhibits the combination of microcellular structures.
- The increase in evaporate time improves the conductive network of composite foams.
- Electromagnetic interference shielding effectiveness (EMI SE) and specific EMI SE of the composite foam displays 69.9 dB and 211.5 dB/(g cm^−3^).
- Polarization, conduction and magnetic loss attenuate microwave energy.

**Supplementary Information:**

The online version contains supplementary material available at 10.1007/s40820-021-00677-5.

## Graphical abstract

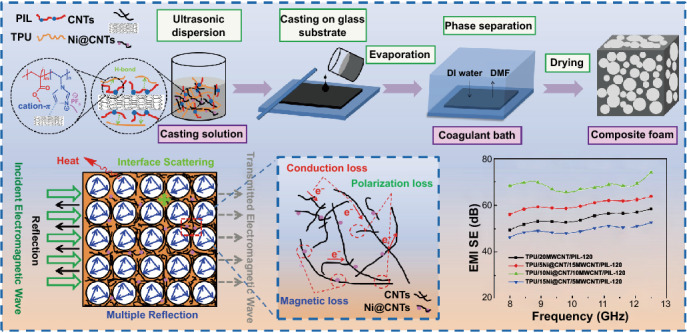

## Introduction

With extensive deployment and development of electronic equipment and communication devices, serious electromagnetic interference (EMI) interrupts the functions of equipment and affects the human organs [1–6]. Recently, more efforts have been made to seek for novel lightweight and high-performance EMI shielding materials [7–12]. Conductive polymer composites (CPCs) containing conductive nanofillers have excellent properties such as lightweight, excellent processability and resistance to corrosion compared with traditional metal-based EMI shielding materials [13–16]. CPCs with porous structures can further lighten the matrix weight and enhance the electrical performance of the composites even at a lower loading of fillers [17–20]. In addition, the preparation methods of electrically CPCs for EMI shielding materials also exert a profound impact on the performance of EMI shielding effectiveness (SE) [21–23].

Phase inversion method, subcritical CO_2_ foaming, freeze-drying, gas foaming, micro-molding and 3D printing have been widely used to prepare EMI shielding materials with porous structures [24–26]. Phase separation methods include non-solvent-induced phase separation (NIPS), thermally induced phase separation (TIPS) and vapor-induced phase separation (VIPS) [27, 28]. Since porous polymer materials can be prepared by phase separation methods, NIPS as the most commonly and widely employed technique can be used to fabricate EMI shielding materials with microcellular structures [29]. In NIPS method, a substrate is covered with a thin layer of polymer solution and then immersed in a non-solvent bath, which causes the phase separation between the polymer-rich and polymer-dilute phases due to thermodynamic instability of the solution [30, 31]. The matrix via gelation and/or solidification can be formed in a polymer-rich phase, whereas the pores can be formed in the polymer-dilute phase [32]. A facile NIPS method can be used to prepare lightweight microcellular polyetherimide (PEI)/graphene foams with a density of 0.3 g cm^−3^ and specific EMI SE of 44 dB/(g cm^−3^) [33].

Multi-walled carbon nanotubes (CNTs), which exhibit an exceptional structure, outstanding mechanical and electrical properties, can provide excellent EMI SE for polymer composite foams [34–36]. However, CNTs are hard to be dispersed in the polymers because of their large specific surface area and strong internal interaction [37, 38]. The modification methods of CNTs include physical noncovalent modification and chemical covalent approaches [39, 40]. For the chemical approaches, the modifier forms the covalent linkage with a skeleton of the CNT by means such as radical polymerization and click chemistry to enhance the dispersion and solubilization of the CNT. However, chemical treatment would damage the translational symmetry of CNT by changing *sp*^2^ carbon atoms to *sp*^3^ carbon atoms, and the properties of nanofillers, including both the electronic and transport properties, are influenced [41]. Noncovalent modification of CNTs such as surfactants, charge-transfer agents and ionic liquids (ILs) is suitable for the facile and mild approaches that may not disrupt the structure and electron characteristics of CNTs [42]. Specifically, CNTs are noncovalently functionalized with modifiers such as cetyltrimethyl ammonium bromide (CTAB), polyvinylpyrrolidone (PVP) and ionic liquid (IL) through π-cation stacking, π-π stacking, H-bonds and hydrophobic interactions. For example, Narayan et al. used PVP as a destacking cum stabilizer of graphene nanosheets. Nonionic surfactant (PVP) adsorbed on the surface of graphene forms a covering layer, thereby preventing contact agglomeration between graphenes. However, the large aspect ratio of CNTs has a certain degree of entanglement, so a stronger force is required to stabilize the dispersion [41, 43]. Poly(ionic liquid)s and polymerizable ionic liquid copolymer (PIL) containing anion-cation pairs have strong cation-π physical interactions with CNTs, which make them promising candidates for noncovalent modification of CNTs [44–47]. PIL-modified CNTs can have a promoted dispersion and increase the electrical conductivity of polymer matrix composites. Compared with other modification technologies of dispersing CNTs in polymer, this method is simpler, more effective and environmentally friendly [48, 49]. For example, Bose et al. reported that imidazolium-based ILs were used to modify noncovalently CNTs through a π-cation stacking interaction. The improved dispersion of IL-CNTs enhanced the electrical conductivity and electromagnetic radiation of polyvinylidene difluoride (PVDF) blends significantly [50].

Thermoplastic polyurethane (TPU) has aroused more interests owing to its high tensile strength and flexibility, good abrasion resistance, excellent chemical and thermal resistance [51–55]. TPU is widely used in flexible displays, smart clothing, electronic textiles and durable elastomeric wheels because TPU mainly consists of hard segments and soft segments [56–58]. The addition of CNTs into TPU can improve the mechanical and electrical properties in the non-conducting matrix for EMI shielding [59, 60]. The lightweight microcellular CPCs are particularly desirable for practical EMI shielding applications. To obtain the lightweight EMI materials, foaming makes the processing procedure easier and improves the EMI shielding performance. For example, Zeng et al. assembled anisotropic porous TPU/CNTs composite foams by the freeze-drying method, and the low-density porous composite with the loading of 76.2 wt% CNTs achieved a SE of about 50 dB [22]. Kim et al. used nickel-coated carbon fiber (NCCF)/CNTs hybrid fillers as conductive fillers to prepare electrically conductive PU composite foams [61]. The EMI SE of PUF/NCCF/CNTs composites was 24.7 dB because NCCF and CNTs synergistically contributed to the conductance and magnetic permeability loss. A lightweight microcellular PVDF composite foam containing 10 wt% Ni-chains showed an enhanced EMI SE (26.8 dB) due to the tailored microcellular structure and high-aspect-ratio magnetic-conductive Ni chains [62]. The metallic nickel nanoparticles and CNTs can synergistically enhance the electrical conductivity, magnetic permeability and dielectric loss of the fillers to the electromagnetic wave.

**Scheme 1:**
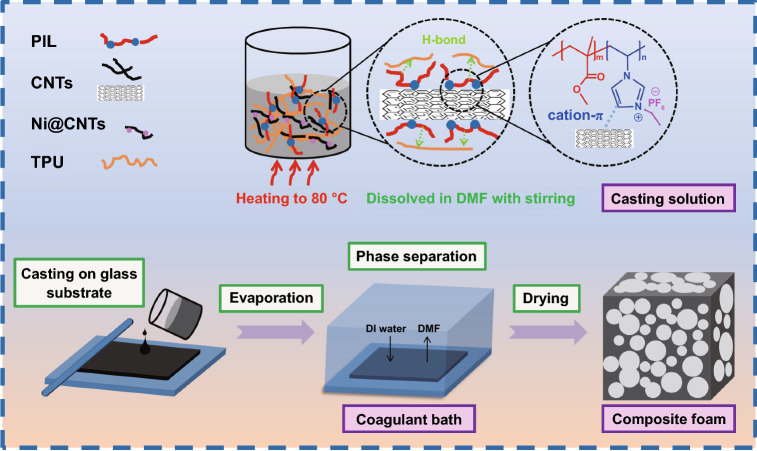
Schematic for the formation of TPU/CNTs composite foams via the NIPS

In this study, CNTs were noncovalently modified with imidazolium-based PIL by the cation-π noncovalent bond interaction and composed with Ni-coated CNT (Ni@CNT) to form hybrid fillers. The TPU nanocomposite foams (TPU/CNTs/Ni@CNT/PIL) with microcellular structures were produced by a non-solvent induced phase separation (NIPS), which was a simple and environmentally friendly method. Scheme 1 shows the formation of TPU nanocomposite foams via the NIPS. The effects of IL content, filler content and evaporation time on the microcellular structure and EMI shielding properties were explored in details. In addition, the mechanism of micropore structure regulation was emphasized, and the influences of micropore structure regulation on the electrical conductivity and the EMI SE were investigated. The EMI shielding mechanism of this system was disclosed considering the microstructures and the network-induced conduction, magnetic loss and polarization loss. This research provides a novel and efficient way to prepare lightweight and high-performance electromagnetic shielding materials.

## Experimental Section

### Materials

TPU particles were purchased from Bayer AG and were polyester-based thermoplastic TPU pellets (790) with a density of 1.21 g cm^−3^. CNTs and Ni@CNTs (10–20 nm in outer diameter, 10–30 μm in length) were supplied by Nanjing XFNANO Materials Tech Co., Ltd., China. *N*,*N’*-dimethylformamide (DMF) and other reagents were provided by Sinopharm Chemical Reagent (China). The synthesis and characterization of PIL are shown in Scheme S1, Figs. S1 and S2.

### Preparation of TPU Nanocomposite Foams via NIPS

The fabrication of TPU nanocomposite foams is schematically illustrated in Scheme 1. Firstly, the TPU and PIL were dissolved in anhydrous DMF with magnetic stirring at 80 ℃ for 2 h. Simultaneously, CNTs and/or Ni@CNTs were dispersed in the solution of PIL with the aid of sonication. The weight ratio of CNTs to PIL was 4 to 1. The suspension of modified CNTs or pristine CNTs was added to the TPU solution, and the mixture was subjected to the ultrasonic treatment for 2 h to form a homogeneous slurry. The resulting slurry was degassed under vacuum and then casted on a pre-cleaned planar glass substrate. DMF was chosen as polymer solvent, whereas deionized (DI) water was used as a non-solvent. The casted film was allowed to evaporate for a certain time (3, 15, 30, 60, 90 and 120 min) before immersed in the coagulation bath to induce phase inversion. The symbol “x” in the TPU/CNTs-x, TPU/CNTs/PIL-x and TPU/CNTs/Ni@CNTs/PIL-x represents the evaporate time (3, 15, 30, 60, 90 and 120 min). This step was crucial for forming a porous structure. Then, the casted film was immersed into the coagulation bath for 6 h. In the casted film, porous structure was formed and solidified by phase separation process through the exchange of solvent (DMF) and non-solvent (water) in the coagulation bath. Finally, the TPU composite foams were obtained after washing with deionized water to remove the residual solvent and drying at 40 ℃ for 24 h. Table 1 shows the formulation of TPU composite foams.

**Table 1:** Formulation of TPU composite foams

Sample	TPU	CNTs	Ni@CNTs	PIL	DMF
TPU	15	–	–	–	85
TPU/10CNTs	13.5	1.5	–	–	85
TPU/15CNTs	12.75	2.25	–	–	85
TPU/20CNTs	12	3	–	–	85
TPU/25CNTs	11.25	3.75	–	–	85
TPU/10CNTs/PIL	13.13	1.5	–	0.38	85
TPU/15CNTs/PIL	12.19	2.25	–	0.56	85
TPU/20CNTs/PIL	11.25	3	–	0.75	85
TPU/25CNTs/PIL	10.31	3.75	–	0.94	85
TPU/15CNTs/5Ni@CNTs/PIL	11.25	2.25	0.75	0.75	85
TPU/10CNTs/10Ni@CNTs/PIL	11.25	1.5	1.5	0.75	85
TPU/5CNTs/10Ni@CNTs/PIL	11.25	0.75	2.25	0.75	85

### Characterization

The Raman spectroscopy was performed with a Raman spectrometer (LABRAM-HR), which is equipped with an excitation wavelength of 532 nm. The XPS spectra of the samples were obtained via an X-ray photoelectron spectroscopy (ESCALAB250Xi, Thermo Fisher). The morphology of sample was observed by a scanning electron microscope (SEM, HITACHI, SU-8020). The density (ρ) of microcellular foams can be calculated by the water displacement method. The electrical conductivity performance was determined using a multi-function digital electric meter by a homemade fixture (Victor Tech, Victor 86-e).

The EMI SE performance of TPU composite foams was measured using a vector network analyzer (N5247A, Agilent Technologies) in X band [63]. The corresponding dimension of samples was 22.86 × 10.16 × 2 mm^3^. The EMI shielding properties of the as-prepared materials were evaluated with S parameters, which were used to calculate SE_T_ (total shielding effectiveness), SE_R_ (reflection loss) and SE_A_ (absorption loss) [28].

## Results and Discussion

### Characterization of CNTs and CNTs/PIL

**Fig. 1:**
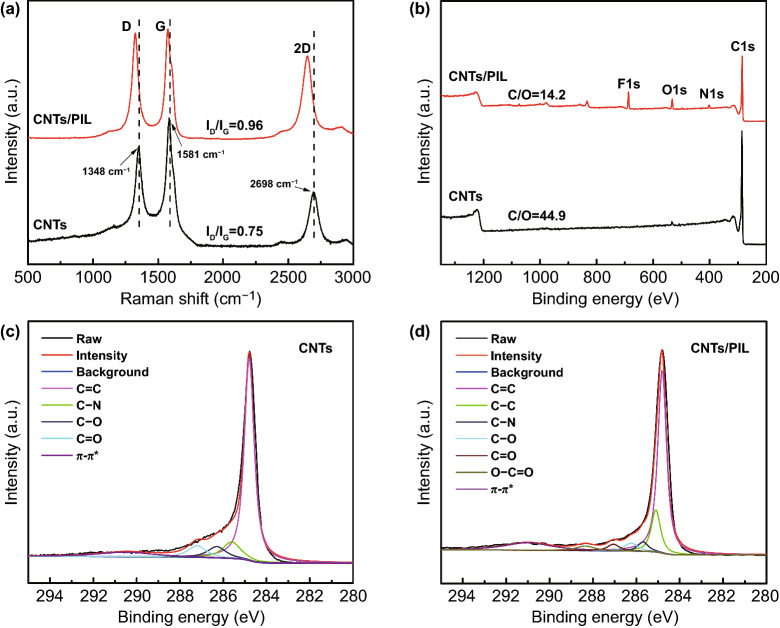
Raman spectra **a** and XPS spectra **b** of CNTs and CNTs/PIL; C 1 s XPS spectra of CNTs **c** and **d** CNTs/PIL

Figure 1a shows the Raman spectra of CNTs and CNTs/PIL hybrids. Pristine CNTs generally show three characteristic peaks. The D-band (1348 cm^−1^) derives from amorphous carbon and lattice defects in the structure, the G-band (1581 cm^−1^) corresponds to the tangential vibrations of carbon atoms, and the 2D-band (2697 cm^−1^) is closely related to the electronic band structure of carbon nanomaterials [38]. The characteristic peaks of D, G and 2D-bond of CNTs/PIL appear at 1324, 1574 and 2650 cm^−1^, respectively. The blue shift of the characteristic peaks indicates that the electron density of CNTs is strengthened owing to the cation-π interaction between imidazolium groups in PIL and π-electrons in CNTs. The increased intensity ratio of *I*_D_/*I*_G_ from 0.75 to 0.96 indicates that the cation-π interaction affects the π–π electronic conjugation and the increase in vibration energy [47].

The C 1 s spectra of CNTs and CNTs/PIL from the XPS analysis are shown in Fig. 1b. As shown in Fig. 1b, the C 1 s signal is reduced in the full scan XPS spectra of CNTs/PIL sample, whereas there is an obvious presence of F ls, O ls and N ls elements because of the modification of CNTs by PIL. As calculated, the elemental concentrations ratio of C and O in the CNTs/PIL is 14.2, which is much lower than that of pristine CNTs (44.9) because of the cation-π interactions [41]. The fitted curves of C 1 s spectra of CNTs and CNTs/PIL are shown in Fig. 1c, d, respectively. These C 1 s spectra can be deconvoluted into various sub-peaks with different binding energies: *sp*^2^ C=C (284.8 eV), *sp*^3^ C–C (285.1 eV), C–N at 285.7 eV, C–O at 286.3 eV, C=O at 287.2 eV, O–C=O at 288.3 eV and π–π* at 290.9 eV. The XPS spectra data are displayed in Tables S1 and S2. Compared with CNTs, the C 1 s peaks of the CNTs/PIL hybrids appear to have two new sub-peaks at around 285.1 and 288.3 eV because the π-π electronic conjugation of pristine CNTs is affected by noncovalent modifications of the PIL [44]. All these results indicate that PIL has successfully modified CNTs by noncovalent cation-π interaction, thereby improving their dispersion and compatibility in the polymer matrix.

### Morphology of TPU Composite Foams

**Fig. 2:**
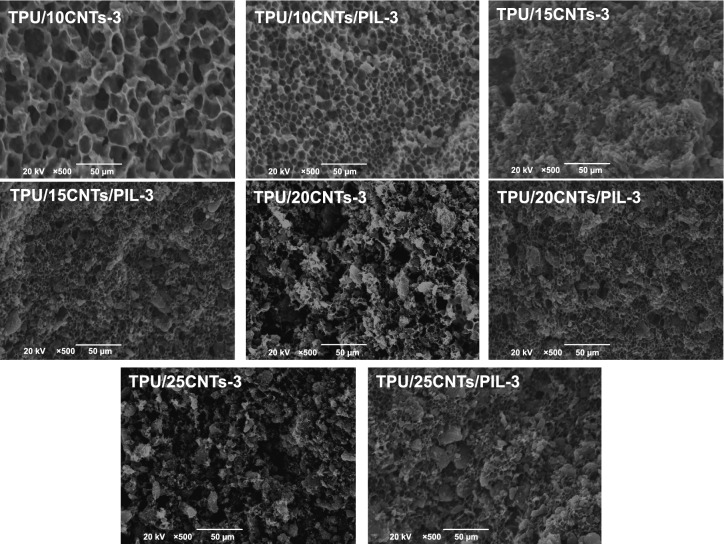
Typical SEM images for fracture morphology of microcellular TPU/CNTs and TPU/CNTs/PIL composite foams with 3 min evaporate time

The typical SEM microstructures shown in Figs. 2 and S3 are used to explore the effect of CNTs modification by PIL on the morphology of TPU composite foams with 3 min evaporate time and different filler contents. The pore size distribution of TPU/10CNTs −3(a) and TPU/10CNT/PIL−3(b) with 3 min evaporate time is shown in Fig. S4 and Table S1. The honeycomb microcellular structure of TPU/CNTs and TPU/CNTs/PIL foams was both well-defined when the filler loading was less than 20 wt% because the diffusion of water to casting solution of the composite can induce physical gelation and phase separation [64]. However, the honeycomb microcellular structure of TPU/20CNTs and TPU/25CNTs foams was destroyed when the filler loading was increased to 20 and 25 wt%, because a higher content of CNTs led to the increased viscosity of the casting solution. After modifying CNTs via PIL, the cell size of TPU/CNTs/PIL composite foams is smaller and the microcellular structure is homogeneous with the same filler content compared with the TPU/CNTs composite foams. The reason for this phenomenon is that PIL decreases the surface tension gradient at the surface of the non-solvent phase and thereby attenuates the Marangoni convection. This in turn decreases the mass-transfer and the size of solvent dilute phase [65]. In addition, the cation-π interaction between PIL and CNTs improved the dispersion of CNTs in the casting solution and increased the heterogeneous nucleation sites. The uniform microcellular structure of TPU/25CNTs/PIL was slightly destroyed because of the higher viscosity of the suspension. The driving force induced by the solvent concentration is affected by the physical barrier of the high content filler, which inhibits the mass-transfer and forms irregular honeycomb microcellular structure [32, 66].

### Electrical and EMI Shielding Properties of TPU Composite Foams

**Fig. 3:**
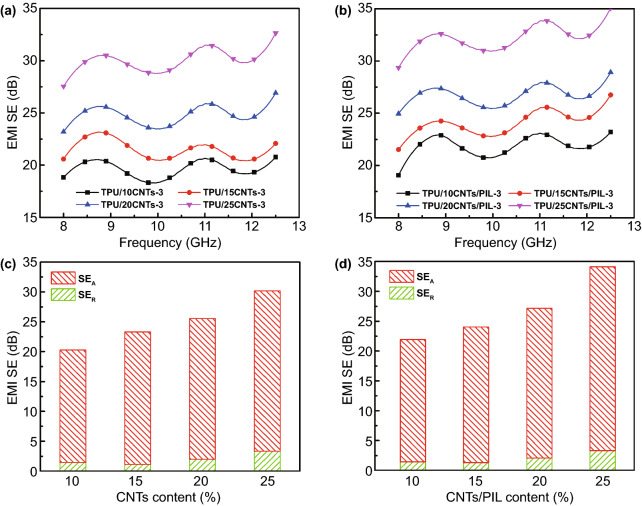
EMI SE of TPU/CNTs **a** and TPU/CNTs/PIL **b** composite foams with 3 min evaporate time and various concentrations; SE_A_ and SE_R_ of TPU/CNTs **c** and TPU/CNTs/PIL **d** composite foams with 3 min evaporate time and various concentrations at 9.0 GHz

The EMI SE of TPU composite foams containing different filler contents with 2 mm thickness and 3 min evaporate time is shown in Fig. 3a, b. The EMI SE exhibits fluctuations with frequency because the microcellular structure in the entire composite material results in the non-uniformity of the discrete conductive network size [34]. The EMI SE value of TPU composite foams increases with increasing the filler content. As the filler content is increased to 20 wt%, the EMI SE of TPU/20CNTs and TPU/20CNTs/PIL foams is improved to 25.5 and 27.2 dB at 9.0 GHz, respectively. By increasing the loading to 25 wt%, the EMI SE value of TPU/25CNTs and TPU/25CNTs/PIL foams reaches 30.2 and 34.1 dB, respectively. The TPU/CNTs/PIL composite foams have a higher EMI SE value because PIL coating on the surface of CNTs reduces the surface energy and enhances the interface between the substrates of TPU [67]. The modification of CNTs by PIL enhances the dispersion of CNTs and increases the viscosity of casting solution in the phase separation process. It provides more heterogeneous nucleation sites and inhibits the aggregation and combination of microcellular structure, which plays a role in regulating the microcellular morphology and reducing the size of microcellular [33, 34].

The SE_T_ is mainly composed of SE_A_, SE_R_ and SE_M_. SE_M_ can be ignored when the SE_T_ is greater than 15 dB [68]. To further illuminate the EMI shielding mechanism of the TPU composite foams, the measured scattering parameters are used to calculate the SE_A_ and SE_R_ at 9.0 GHz as shown in Fig. 3c, d. Clearly, the SE_A_ and SE_R_ of composite foams increase with increasing the filler content. In addition, the SE_R_ occupies a small proportion of the SE_T_ and the SE_A_ is the main EMI shielding mechanism in the X-band for TPU composite foams. The multiple reflections of electromagnetic waves caused by the microcellular structure and conductive network in the foams converted the microwave power into heat dissipation [52]. The absorption efficiency of TPU/CNTs/PIL is slightly higher than that of TPU/CNTs. The absorption originates from the electrical conductivity of CNTs, and polarization loss between TPU substrates and conductive fillers. It can be considered that the smaller microcellular structure and more uniformly dispersed CNTs enhance the multiple reflection and energy conversion through conductivity and polarization loss [69].

### Effect of Evaporate Time on Morphology of TPU Composite Foams

**Fig. 4:**
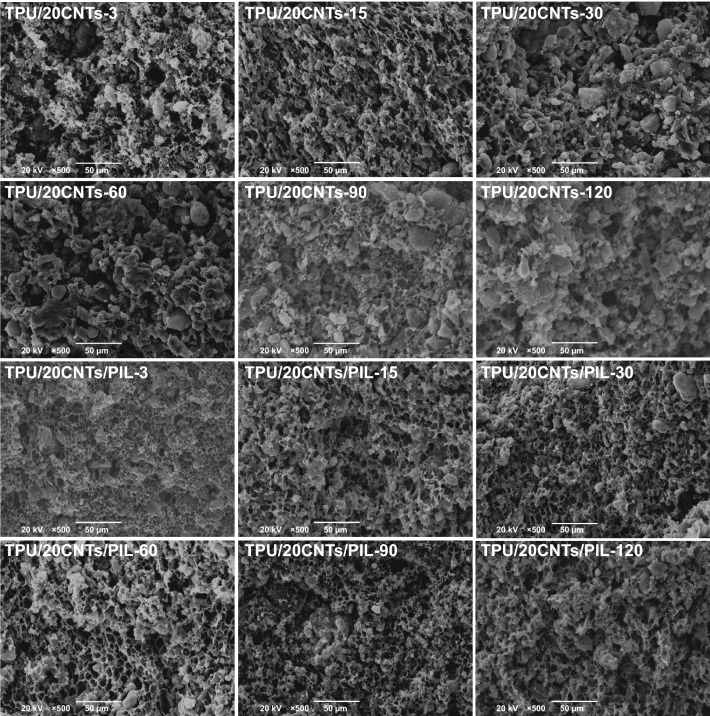
SEM images of TPU/20CNTs and TPU/20CNTs/PIL composite foams with different evaporate time (3, 15, 30, 60, 90 and 120 min)

Evaporate time refers to the time interval between the casting solution pouring into the mold and immersing it in the coagulation bath. The effect of evaporate time on the kinetics and thermodynamic equilibrium of phase separation process was revealed by SEM. As shown in Figs. 4 and S5, the morphology of composite foams gradually becomes dense with the increase in the evaporation time. In contrast, the morphology of TPU/20CNTs is rough and has many voids because of the increased viscosity and the introduced physical barrier of CNTs. The microcellular structure of PU/CNT composite is almost completely destroyed when the evaporation time exceeds 30 min. But the microcellular structure of TPU/20CNTs/PIL composites is not destroyed and becomes more uniform compared with that of TPU/20CNTs [53].

The morphological differences can be attributed to the effects of PIL and evaporation time during the NIPS process. Firstly, PIL-modified CNTs improved the dispersion of CNTs, acted as nucleation agents and provided numerous nucleation sites for the NIPS foaming process. Subsequently, PIL as a surfactant can be dissolved in the solvent, decreases the surface tension of casting solution, and then the agglomeration of DI-rich droplets at the liquid/air interface is hindered [30]. Finally with increasing the evaporate time, the viscosity of casting solution is increased and the formed physical barrier leads to the cell agglomeration.

### Effect of Evaporate Time on Electrical and EMI Shielding Properties

**Fig. 5:**
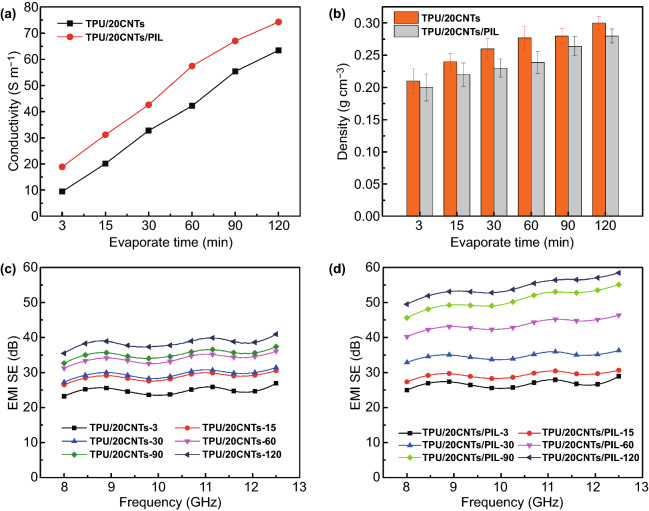
Conductivity **a** and density **b** of TPU composite foams with different evaporate time; **c** and **d** EMI SE of TPU composite foams

Figure 5a compares the electrical conductivity of TPU/CNTs and TPU/CNTs/PIL composite foams with different evaporate time. It can be seen that the electrical conductivity of TPU/20CNTs/PIL-3 (18.9 S m^−1^) is 2 times that of TPU/20CNTs-3 (9.4 S m^−1^). The electrical conductivity of TPU/20CNTs/PIL-15 (31.2 S m^−1^) is 1.5 times that of TPU/20CNTs-15 (20.1 S m^−1^). When the evaporation time exceeds 30 min, the electrical conductivity of the TPU/20CNTs and TPU/20CNTs/PIL is increased slowly and is of the same order of magnitude. It can be found that the electrical conductivity of TPU/20CNTs/PIL is higher than that of TPU/20CNTs. It is indicated that the conductive network in the composites is gradually improved due to the improved dispersion and distribution of the conductive fillers with increasing the evaporation time. These proved that PIL is helpful to improve the dispersion of CNTs through the noncovalent modification. With the extension of evaporation time, the solid–liquid phase separation induced the conductive filler to be distributed in the rich polymer phase, which further improved the conductive network of composite foams and finally reached saturation. In summary, the noncovalent modification of CNTs by PIL and the distribution of conductive fillers regulated by solid–liquid phase separation have significantly improved the electrical conductivity of the foams [70].

The densities of the TPU/20CNTs and TPU/20CNTs/PIL composite foams with different evaporate time are shown in Fig. 5b. It can be seen that the density of both composite foams increases with the extension of evaporate time. It should be noted that the modification of CNTs by PIL significantly decreases the density of composite foams. The presence of PIL optimizes the dispersion of CNTs and increases the viscosity of the casting solution. This in turn results in uniform cells and reduces mass transfer and density of the composite foam [58].

Figure 5c, d shows the EMI SE of the TPU/20CNTs and TPU/20CNTs/PIL foams. It is seen that the EMI SE of TPU/CNTs and TPU/CNTs/PIL is gradually increased as the evaporation time is increased from 3 to 120 min. The conductive network is improved with the increase in micro-phase separation, which provides more pathways for the transition and transmission of free electrons. The results indicate that the solid–liquid phase separation improves the conductive network of composite foams and reduces the microcellular size, which strengthens the multiple reflection of electromagnetic wave [71]. In addition, compared with the TPU/CNTs composite foams, the addition of PIL in TPU/CNTs/PIL had given a higher EMI SE value under the same condition. The maximum EMI SE of TPU/20CNTs/PIL reaches 53.3 dB because the PIL improves the dispersion of CNTs and provides more transmission of free electrons and stronger interfacial polarization. Uniform microcellular structure and perfect conductive network had a large number of electrons or holes acting as carriers of mobile charges [28]. Under the action of external electromagnetic waves, these carriers are transmitted along the conductive network and then convert the EM energy into the thermal energy.

**Fig. 6:**
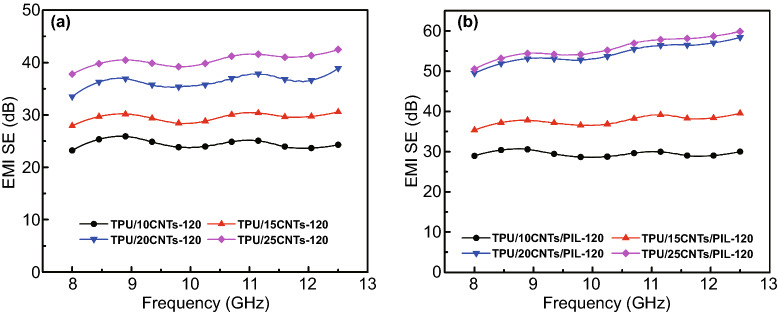
EMI SE of TPU/CNTs and TPU/CNTs/PIL composite foams with various filler concentrations and an evaporate time of 120 min

In order to further illuminate the influences of filler content and evaporate time on the EMI SE of composite foams, various filler concentrations were investigated when the evaporate time was fixed at 120 min. The results are shown in Fig. 6. The EMI SE values of TPU/CNTs and TPU/CNTs/PIL composite foams are still positively correlated with the filler concentration. The EMI SE of TPU/25CNTs and TPU/25CNTs/PIL composite foams is 40.6 and 54.6 dB, respectively, when the filler content is 25 wt%. The EMI SE of composite foams is increased significantly with the increase in filler concentration. However, the EMI SE of TPU/CNTs/PIL composite foam is stabilized at 60 dB without any significant increase when the filler concentration is beyond 20 wt%. This is because the conductive network has been substantially improved when the filler concentrations are 20 wt%. The further increase in filler concentration has a weak strengthening effect on the conductive network and may even destroy the microcellular structure of the composite foams [72]. These results are consistent with that from SEM images (Fig. 2). Therefore, the EMI shielding performance of composite foams mainly originates from the formed interconnected CNTs networks and the porous structure that attenuate the incident electromagnetic waves via multiple reflections [73].

**Fig. 7:**
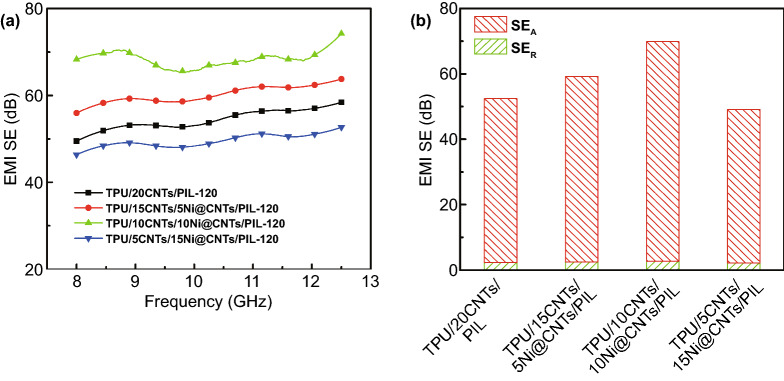
**a** EMI SE of TPU/CNTs/Ni@CNTs/PIL foams; **b** comparison of SE_T_, SE_A_ and SE_R_ of TPU/CNTs/Ni@CNTs/PIL foams with 120 min evaporate time and various concentrations at 9.0 GHz

The EMI SE of TPU/CNTs/Ni@CNTs/PIL foams with 2.0 mm thickness are shown in Fig. 7a. The EMI SE of TPU/CNTs/Ni@CNTs/PIL foams is firstly increased and then reduced with increasing the Ni@CNTs content. The electrical conductivity of TPU/15CNTs/5Ni@CNTs/PIL, TPU/10CNTs/10Ni@CNT/PIL and TPU/5CNTs/15Ni@CNTs/PIL foams with 3 min are 15.3, 12.2 and 8.7 S m^−1^, respectively. The electrical conductivity of TPU/20CNTs/PIL with 3 min is 18.9 S m^−1^. The electrical conductivity of TPU/15CNTs/5Ni@CNTs/PIL, TPU/10CNTs/10Ni@CNT/PIL and TPU/5CNTs/15Ni@CNTs/PIL foams with 120 min is 65.3, 56.8 and 43.7 S m^−1^, respectively. The electrical conductivity of TPU/20CNTs/PIL with 120 min is 74.2 S m^−1^. The electrical conductivity of PU/CNTs/Ni@CNTs/PIL foams with the same filler content is slightly lower than that of PU/20CNTs/PIL because the electrical conductivity of CNTs is slightly lower than that of Ni@CNTs. The EMI SE of TPU/10CNTs/10Ni@CNTs/PIL foam is 69.9 dB at 10 wt% Ni@CNTs and 10 wt% CNTs content (Fig. S6 shows the Raman spectra and XPS spectra of CNTs/Ni@CNTs/PIL, Tables S1 and S2 show the date of XPS spectra). The EMI SE of TPU/20CNTs/PIL foam is slightly lower than that of TPU/10CNTs/10Ni@CNTs/PIL foam.

Figure 7b shows the SE_T_, SE_R_ and SE_A_ at 9 GHz of all foams. It is indicated that the absorption mainly contributes to the EMI SE for all foams. The multi-pore microstructure of the foams effectively relieves the impedance mismatching between air and absorber when the electromagnetic wave propagates to the foams. Thus, the electromagnetic wave easily radiates into the interior of foams, resulting in a low reflection on the surface. Then, the entered electromagnetic microwaves are reflected among these micropores and their walls in the composite foams, which is converted into heat energy to be dissipated [74]. The conductive linkages of CNT/Ni@CNT/PIL act as an inter-transporting charge carriers for tunneling more electrons and also enhancing the hopping mechanism within the shielded foam material [75]. The abundant terminal groups and the large number of charge carriers of CNT/Ni@CNT/PIL could interact with the incident waves and dissipate them by converting to heat [76]. In addition, the polarization loss, which was caused by interfacial polarization between TPU substrates and conductive fillers, conduction loss caused by conductive network of fillers and magnetic loss caused by Ni@CNTs, synergistically attenuate the microwave energy. Therefore, these results confirm that SE_A_ plays a primary role in the SE_T_ of composite foams.

**Fig. 8:**
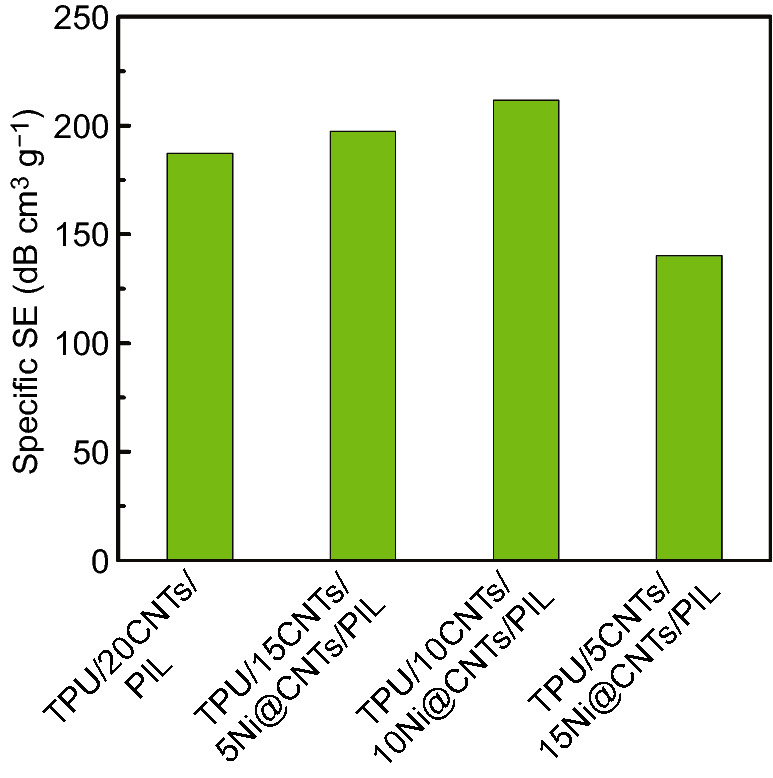
Specific EMI SE of TPU/CNTs/Ni@CNTs/PIL composite foams with various filler concentrations and the evaporate time of 120 min at 9.0 GHz

Figure 8 shows the specific EMI SE (i.e., EMI SE divided by the sample density and thickness, SSE) of TPU/CNTs/Ni@CNTs/PIL with various filler concentrations and an evaporate time of 120 min at 9 GHz. The densities of TPU/15CNTs/5Ni@CNTs/PIL, TPU/10CNTs/10Ni@CNTs/PIL and TPU/5CNTs/15Ni@CNTs/PIL composites are 0.30, 0.33 and 0.35 g cm^−3^, respectively. The density of TPU/10CNTs/10Ni@CNTs/PIL increases with increasing the Ni@CNTs loading. The morphology of composite gradually becomes dense with the increase in the Ni@CNTs content (Fig. S7) because Ni@CNTs are easier to agglomerate than CNTs and the density of Ni@CNTs is greater than that of CNTs. The compressive strength of TPU/CNTs/Ni@CNTs/PIL is slightly decreased with increasing the Ni@CNTs content (Figs. S8 and S9). The highest specific EMI SE of TPU/20CNTs/PIL and TPU/10CNTs/10Ni@CNTs/PIL reaches up to 187.2 and 211.5 dB/(g cm^−3^), respectively.

**Fig. 9:**
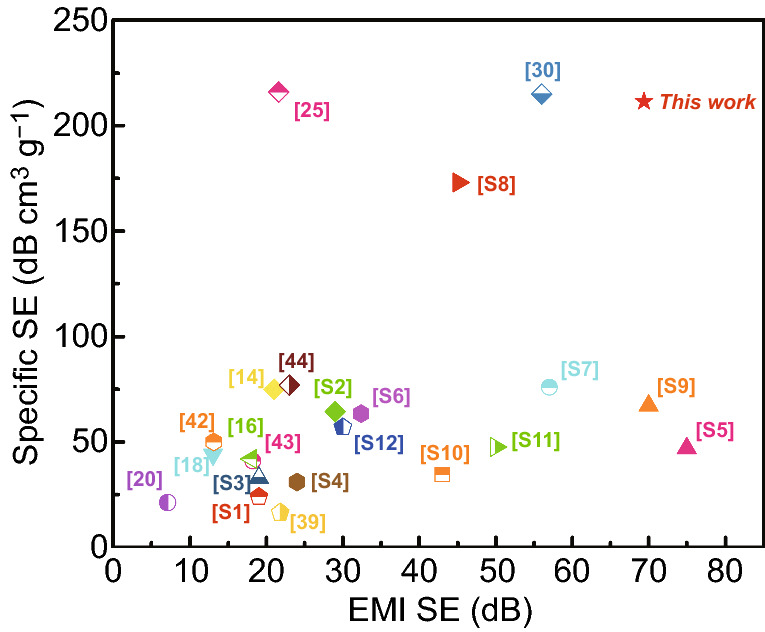
Comparison of EMI shielding performance of various composite foams ever reported

**Fig. 10:**
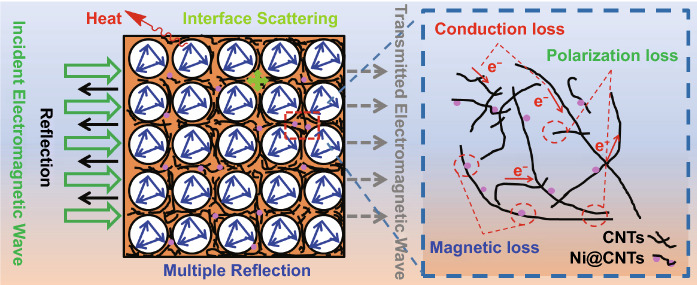
Schematic description of the microwave transfer across the TPU composite foams

Figure 9 summarizes the EMI SE and SSE of the foams-based EMI shielding materials reported in the literatures. Table S4 shows the detailed results of the EMI performance. It is indicated that the TPU/10CNTs/10Ni@CNTs/PIL foams show a very high SSE (211.5 dB cm^3^ g^−1^). The high SE and SSE for the foams derive from its particular microcellular structure, which is schematically illustrated in Fig. 10. The TPU/10CNTs/10Ni@CNTs/PIL foam has high electrical conductivity and magnetic permeability [63]. These CNTs possess large specific surface areas and high electrical conductivity. PIL-modified CNTs can wet and interact with PU matrix, which are essential for interfacial polarization and re-reflections. The magnetic loss is due to the effect of magnetic filler Ni@CNT, which introduce more and more magnetic dipoles into the PU matrix. In addition, the uniform dispersion of CNTs and Ni@CNTs increases the interface area between fillers and TPU matrix, which increases the interface polarization and causes the interface polarization dielectric loss [77]. The small-sized microcellular structure adds extensive interfaces and enhances the attenuation of incident electromagnetic waves via multiple reflections [78]. Thus, the composite foams suggest a great potential in applications for aircraft and spacecraft field, which has the characteristics of lightweight and efficient specific EMI SE.

## Conclusion

Lightweight microcellular TPU/CNTs/Ni@CNTs/PIL composite foams with high EMI SE are fabricated with an environmentally benign and efficient NIPS technique. TPU/CNTs/PIL composite foams have a higher EMI SE value. This is attributed to the PIL coating on the surface of CNTs, which reduces the surface energy and increases the interface areas between the TPU substrates and the fillers. Moreover, the evaporate time regulates the microcellular structure and obviously improves electrical conductivity and EMI shielding properties. The EMI SE of TPU/10CNTs/10Ni@CNT/PIL foam is 69.9 dB compared with TPU/20CNTs/PIL (53.3 dB) at the same CNTs content. The highest specific EMI SE of TPU/20CNTs/PIL and TPU/10CNTs/10Ni@CNTs/PIL reaches up to 187.2 and 211.5 dB/(g cm^−3^), respectively. The corresponding conduction and magnetic loss, polarization loss in the TPU/10CNTs/10Ni@CNTs/PIL nanocomposites attenuate the electromagnetic waves. The composite foams, which have the characteristics of lightweight and efficient specific EMI SE, show great potential applications in aviation and electronic industrial field.

## Supplementary Information

Below is the link to the electronic supplementary material.

Supplementary file1 (PDF 1077 kb)
