# Supplementary material for: Interface Engineered Microcellular Magnetic Conductive Polyurethane Nanocomposite Foams for Electromagnetic Interference Shielding
Source: Nanomicro Lett. 2021 Jul 8;13:153. doi: 10.1007/s40820-021-00677-5 (PMC8266988; doi:10.1007/s40820-021-00677-5)
Supplement: Supplementary file 1 — Supplementary file1 (PDF 1077 kb) [file 40820_2021_677_MOESM1_ESM.pdf]

Supporting information for

Interface Engineered Microcellular Magnetic Conductive Polyurethane Nanocomposite Foams for Electromagnetic Interference Shielding

Guolong Sang[1], Pei Xu[1, *], Tong Yan[1], Vignesh Murugadoss[2, 3], Nithesh Naik[4], Yunsheng Ding[1], Zhanhu Guo[3, *]

[1]Department of Polymer Science and Engineering, School of Chemistry and Chemical Engineering, and Anhui Key Laboratory of Advanced Functional Materials and Devices, Hefei University of Technology, Hefei 230009, P. R. China

[2]Advanced Materials Division, Engineered Multifunctional Composites (EMC) Nanotech. LLC, Knoxville, TN 37934 USA

[3]Integrated Composites Laboratory (ICL), Department of Chemical and Biomolecular Engineering, University of Tennessee, Knoxville, TN, 37996, USA

[4]Department of Mechanical & Manufacturing Engineering, Manipal Institute of Technology, Manipal Academy of Higher Education, Manipal-576104, Karnataka, India

*Corresponding authors. E-mail: chxuper@hfut.edu.cn (P. Xu), zguo10@utk.edu (Z. Guo)

Supplementary Information

S1 Synthesis of PIL

Methyl methacrylate (MMA), 1-vinylimidazole (IVm), azodiisobutyronitrile (AIBN), *1*-bromoethane (EtBr), potassium hexafluorophosphate (KPF_6), diethyl ether (DEE) and other reagents were all analytically pure supplied by Sinopharm Chemical Reagent (China) and used as received without any further purification.

The reaction procedure of PIL is shown in Scheme S1. First, MMA (2.6 g) and IVm (2.5 g) were completely dissolved in DMF (25 mL) by stirring for 1 h at room temperature under nitrogen atmosphere. Then, catalyst AIBN (40 mg) was slowly added into the mixture and the polymerization reaction was allowed to last for 24 h at 65 ℃ with stirring. After the reaction, the resultant was cooled down to 45 ℃ and a slight excess of EtBr (2.5-3.0 g) was added for ion-solvating reaction under nitrogen flow for 24 h to fully react. In order to isolate the sample in the solid state, precipitation was carried out using diethyl ether as a non-solvent. The resultant was added dropwise into diethyl ether and white precipitate appeared with continuous stirring. On completion, precipitate was taken out and dried at 110 ℃ for 24h to remove ether and DMF to give the product P[MMA-VEIm][Br] as a solid. Finally, P[MMA-VEIm][Br] (2.5 g) and slight excess of KPF_6 (6.85 g) were dissolved in 40 mL deionized water, respectively. The KPF_6 aqueous solution was added dropwise to the P[MMA-VEIm][Br] solution with continuously stirring. A few minutes later, white flocculent precipitate was settled and then reacted at 30 ℃ for 24 h to ensure the complete ion exchange of PF^{6-} and Br^-. The precipitate was washed with a copious amount of deionized water and dried under vacuum for 24 h at 75 ℃ to obtain PIL.

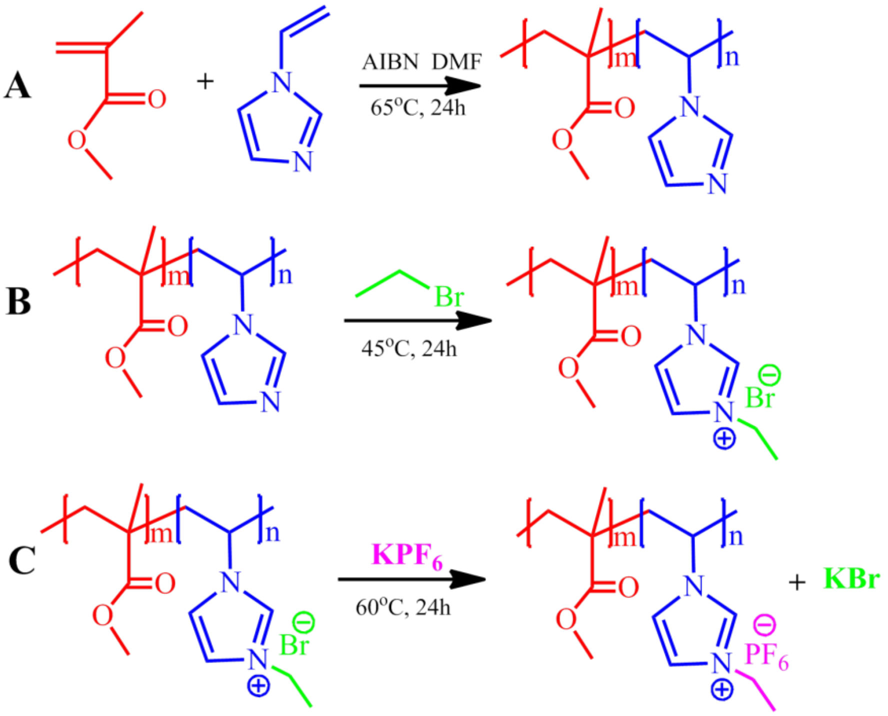

Scheme S1 Preparation methods of the PIL

The expected P[MMA-VEIm][PF_6] copolymer (PIL) was verified by the characterization of chemical structure by ^{1}H NMR and FT-IR. The FT-IR spectra and ^{1}H NMR spectra were performed by Nicolet Nexus 670 and Agilent VNMRS600.

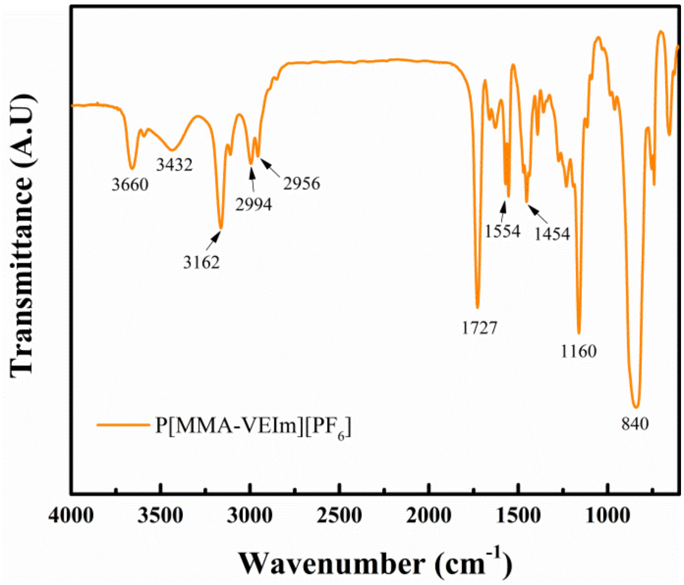

Fig. S1 FT-IR spectra of P[MMA-VEIm][PF_6] (PIL) copolymer

Figure S1 displays the FT-IR spectra in the wavenumber region from 4000 to 500 cm^{-1} of P[MMA-VEIm][PF_6]. The characteristic peaks of 3660 and 840 cm^{-1} are appeared and attribute to the CH…F hydrogen bonding between the [BF_6] anion and hydrogen of the imidazolium ring, which indicated the complete ion exchange. The bands at 3432 cm^{-1} and 1727 cm^{-1} can be assigned to the O-H stretching vibrations. The bands at 1554 and 1454 cm^{-1} are attributed to the C=N stretching modes for the imidazole units. The sharp peaks at 2994 and 2956 cm^{-1} are associated with CH_2 stretching, while the band at 1160 cm^{-1} is assigned to an inplane bending vibration of C–H, which was formed during protonation.

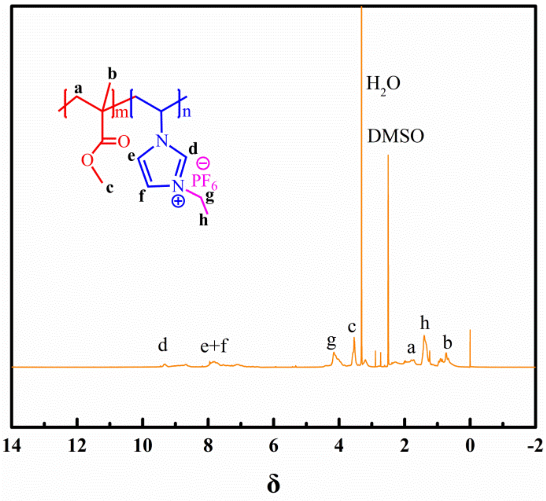

Fig. S2 ^{1}H NMR spectra of PIL copolymer

^{1}H NMR (600 MHz, DMSO), δ (TMS, ppm) spectra are consistent with the study: 9.34 (1H, m, –N–CH–N–), 7.82 (2H, m, –N–CH–CH–N–), 4.17 and 1.41 (2H and 3H, m, $–N–CH_2–CH_3$), 3.54 (3H, m, $–COOCH_3$), 0.74 (3H, s, $–CH_3$), 1.75 (2H, m, $–CH_2–$).

S2 Foam Morphology of TPU Foam and TPU Composites Foam

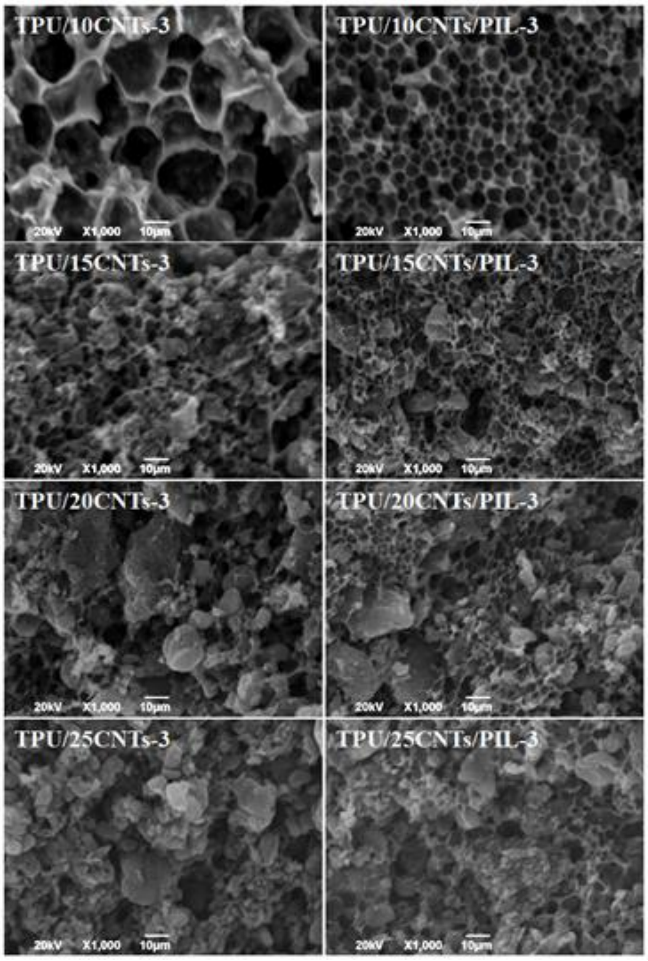

Fig. S3 Typical SEM images for fracture morphology of microcellular TPU/CNTs and TPU/CNTs/PIL composite foams with 3 min evaporate time

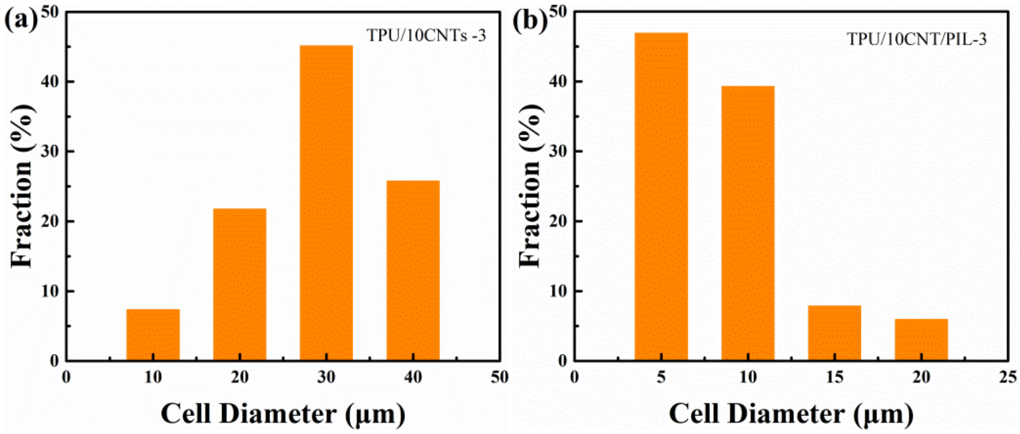

Fig. S4 Pore size distribution of (**a**) TPU/10CNTs -3 and (**b**) TPU/10CNT/PIL-3 with 3 min evaporate time

Table S1 Density of TPU/CNTs and TPU/CNTs/PIL composites with various filler concentrations with 3 min evaporate time

Filler content (wt %)	TPU/CNTs (g/cm^3)	TPU/CNTs/PIL (g/cm^3)
0	0.24	0.23
10	0.17	0.15
15	0.20	0.18
20	0.21	0.20
25	0.26	0.23

The pore size distribution of TPU/10CNTs -3(a) and TPU/10CNT/PIL-3 (b) with 3 min evaporate time is shown in Fig. S4. Because of the limitation to the obtainable foam morphology, we were not able to investigate the foam structure of all samples. Fig. S4 is the microscopic morphology of TPU foam, finger-like pore can be seen in low magnification. The heterogeneous nucleation of the uniformly dispersed fillers in the TPU matrix optimizes the cell size distribution. As can be seen from Table S1, the densities of TPU/CNTs and TPU/CNTs/PIL composites are both lower than that of TPU foam. The density of TPU composites shows an increasing trend with the increase of filler content.

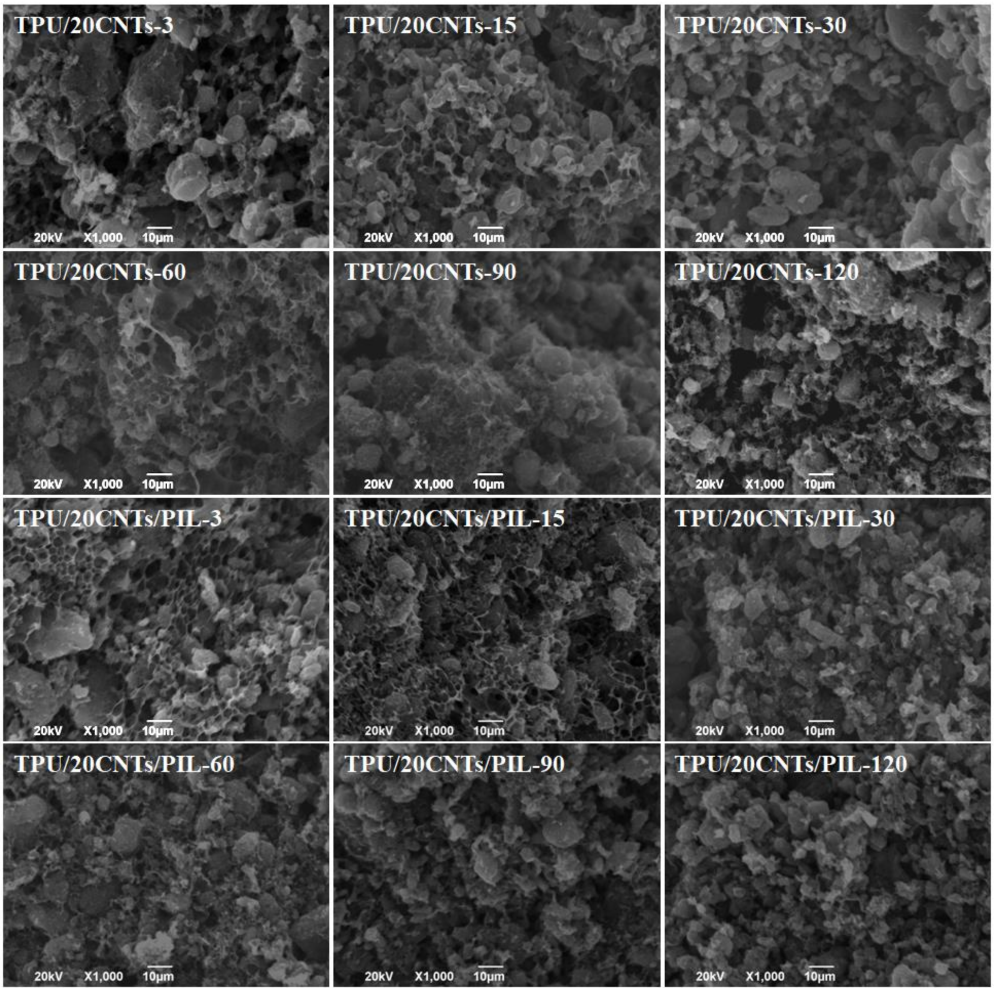

Fig. S5 SEM images of TPU/20CNTs and TPU/20CNTs/PIL composite foams with different evaporate time (3, 15, 30, 60, 90 and 120 min)

S3 Characterization of CNTs/Ni@CNTs/PIL

Figure S6a shows the Raman spectra of CNTs/Ni@CNTs/PIL hybrids with the wavelength of 532 nm. It can be seen that the characteristic peaks of D, G and 2D-bond of CNTs/Ni@CNTs/PIL appear at 1439, 1581, and 2699 cm^{-1}, respectively. The blue shift of the characteristic peaks indicates that the electron density of CNTs and Ni@CNTs is strengthened due to the cation-π interaction between imidazolium groups and π-electrons. The increased value of I_D/I_G to 1.13 indicates a great increase of the defects that are attributed to the cation-π interaction, which affects the π-π electronic conjugation and the increase of vibration energy.

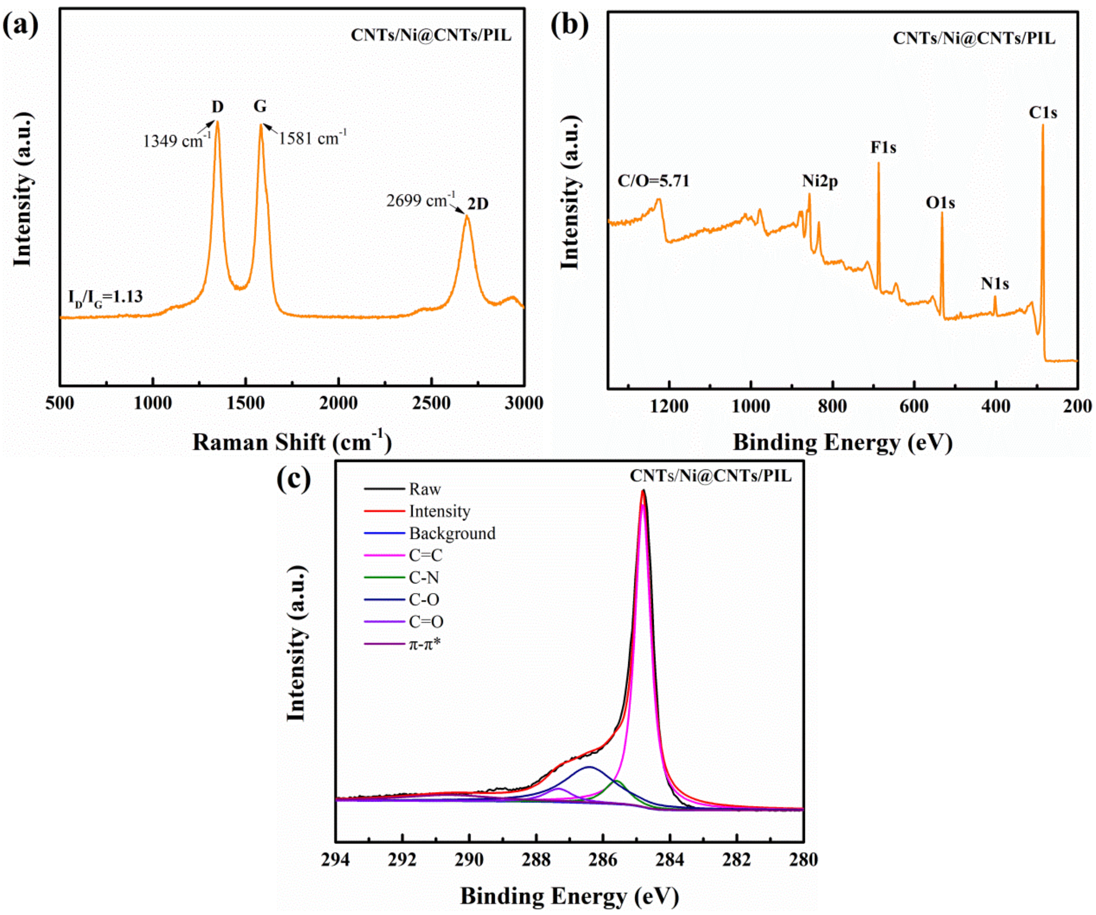

Fig. S6 (**a**) Raman, (**b**) XPS and (**c**) C1s XPS spectra of CNTs/Ni@CNTs/PIL

Table S2 Elemental composition by XPS analysis (atom%)

Sample	C	O	N	F	Ni	C/O
CNTs	96.06	2.14	1.80	–	–	44.9
CNTs/PIL	83.09	6.76	4.11	6.04	–	14.2
CNTs/Ni@CNTs/PIL	78.21	13.66	4.43		3.71	5.71

As shown in the full scan XPS spectra (Fig. S6b), the characteristic peaks of F 1s and Ni 2p appear at 687 and 857 eV, respectively. Figure S6c shows the fitted curves (C 1s peak) of CNTs/Ni@CNTs/PIL hybrids, which can be mainly deconvoluted into five subpeaks at 284.8, 285.6, 286.4, 287.1, and 290.7 eV, assigned to C=C (sp^2), C–N, C–O, C=O, and π-π* shake up, respectively. The XPS spectrum data of the elemental composition and distribution of functional groups of CNTs, CNTs/PIL and CNTs/Ni@CNTs/PIL hybrids are displayed in Table S2 and S3.

Table S3 XPS spectrum data of the distribution of functional groups

Relative atomic percentage [%] (fitting of the C1s peak [eV])							
Sample	C=C	C–C	C–N	C–O	C=O	O–C=O	π-π* shake up
CNTs	284.8	–	285.6	286.3	287.2	–	290.9
CNTs/PIL	284.8	285.1	285.7	286.2	287.1	288.3	291.0
CNTs/Ni@CNTs/PIL	284.8	–	285.6	286.4	287.1	–	290.7

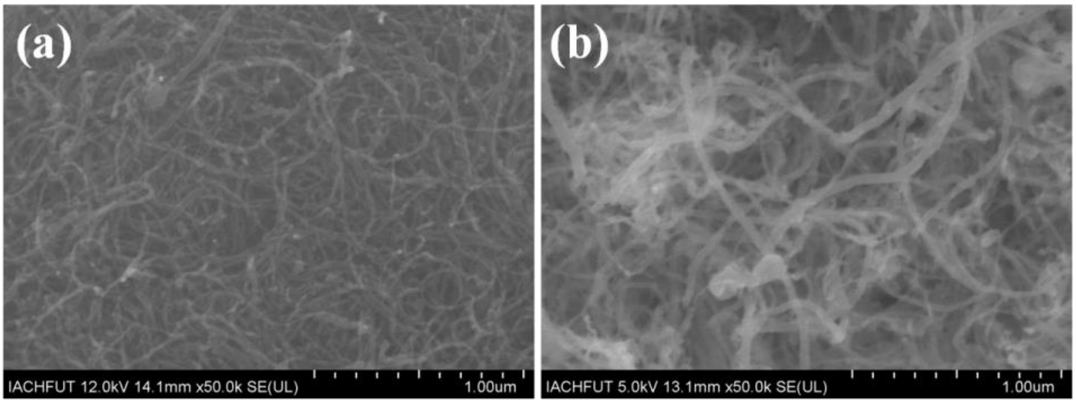

Fig. S7 SEM image of (**a**) CNT and (**b**) Ni@CNT

The structure of CNTs and Ni@CNTs is shown in Fig. S7. It can be seen from Fig. S7b that the surface of Ni@CNTs is rough.

S4 Morphology and Mechanical Properties of TPU/CNTs/Ni@CNTs/PIL Foams

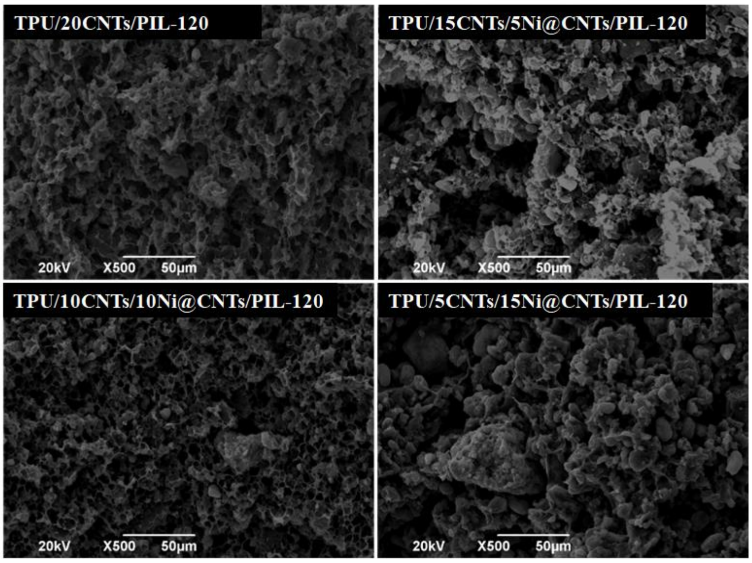

Fig. S8 SEM images of TPU/CNTs/Ni@CNTs/PIL foams with 120 min

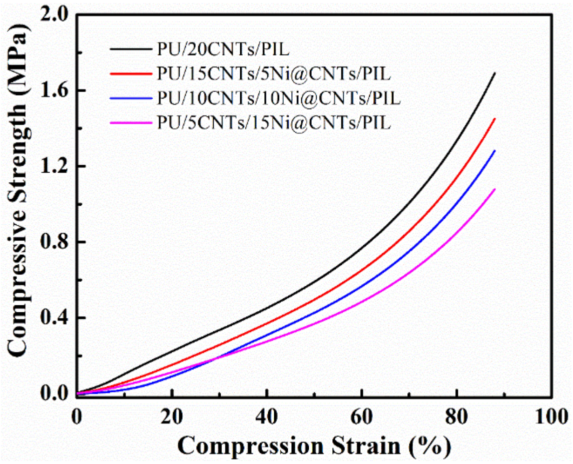

Fig. S9 Mechanical properties of TPU/CNTs/Ni@CNTs/PIL composite foams with various filler concentrations and the evaporate time of 120 min

Figure S8 shows the SEM images of TPU/CNTs/Ni@CNTs/PIL foams with 120 min. Figure S9 shows the mechanical properties of TPU/CNTs/Ni@CNTs/PIL with various filler concentrations and the evaporate time of 120 min. The density of TPU/10CNTs/10Ni@CNTs/PIL increases with increasing Ni@CNTs loading. The morphology of composite gradually become dense with the increase of the Ni@CNTs content because Ni@CNTs are easier to agglomerate than CNTs and the density of Ni@CNTs is greater than that of CNTs. So the compressive strength of TPU/CNTs/Ni@CNTs/PIL is slightly decreased with increasing Ni@CNTs content. When the strain is 80%, the compressive strength of the TPU/20CNTs/PIL and TPU/10CNTs/10Ni@CNTs/PIL foams was 1.69 and 1.28 MPa respectively.

S5 Comparison of EMI Shielding Performance for Various Composite Foams

The shielding effectiveness (SE) and specific SE (SSE) (defined as the SE per unit volume) of the foams based EMI shielding material reported in the literatures are summarized in Table S4.

Table S4 Comparison of EMI shielding performance for various composite foams

Matrix	Filler	Content (wt%)	Thickness (mm)	Density (g/cm^3)	EMI SE (dB)	Specific EMI SE $dB/(g/cm^3)$	Refs.
PMMA	Graphene	5	2.4	0.79	19	24	[S1]
PS	Graphene	30	2.5	0.45	29	64.4	[S2]
PS	CNT	7	1.2	0.56	19	33	[S3]
Paper	Fe_3O_4/Graphene		0.3	0.78	24	31	[S4]
Paper	MCMB/Fe_3O_4		2.5	1.6	75	47	[S5]
Phenolic	MWCNTs	60.6	0.14	0.51	32.4	63.5	[S6]
PVDF	MWCNT	15	2.0	0.79	57	76	[S7]
PS	Graphene	7	2.5	0.26	45.1	173	[S8]
PEDOT	Graphene	25	0.8	1.04	70	67.3	[S9]
PC	MWCNT	20	2.0	1.13	43	34.5	[S10]
ABS	MWCNT	15	1.1	1.05	50	47.6	[S11]
PS	MWCNT	20	2.0	0.53	30	57	[S12]
PI	rGO	16	0.8	0.28	21	75	[14]
PEI	G@Fe_3O_4	10	2.5	0.4	18	42	[16]
PEI	Graphene	10	2.3	0.3	13	44	[18]
Epoxy	MWCNT	3	2.8	0.33	7.1	21.3	[20]
scPLA	CNT	30	3.7	0.1	21.6	216	[25]
Paper	MCMB-MWCNTs	25	0.6	0.26	56	215	[30]
TPU	RGO	6.5	1.8	0.8	21.8	16.6	[39]
PMMA	CNTs @Fe_3O_4	7	2.5	0.38	13.1	50	[42]
PI	RGO/MWCNTs	8	0.5	0.44	18.2	41	[43]
PLLA	MWCNTs	10	2.5	0.3	23	77	[44]
TPU	10Ni-CNT/10CNT/PIL	20	2.0	0.33	69.8	211.5	This work

Supplemenary References

[S1]H.B. Zhang, Q. Yan, W.G. Zheng, Z.X. He, Z.Z. Yu, Tough graphene-polymer microcellular foams for electromagnetic interference shielding. ACS Appl. Mater. Interfaces **3,** 918–924 (2011). https://doi.org/10.1021/am200021v

[S2]D.X. Yan, P.G. Ren, H. Pang, Q. Fu, M.B. Yang et al., Efficient electromagnetic interference shielding of lightweight graphene/polystyrene composite. J. Mater. Chem. **22,** 18772–18774 (2012). https://doi.org/10.1039/C2JM32692B

[S3]Y.L. Yang, M.C. Gupta, K.L. Dudley, R.W. Lawrence, Novel carbon nanotube-polystyrene foam composites for electromagnetic interference shielding. Nano Lett. **5,** 2131-2134 (2005). https://doi.org/10.1021/nl051375r

[S4]W.L. Song, X.T. Guan, L.Z. Fan, W.Q. Cao, C.Y. Wang et al., Magnetic and conductive graphene papers toward thin layers of effective electromagnetic shielding. J. Mater. Chem. A **3,** 2097−2107 (2015). https://doi.org/10.1039/D1TA00890K

[S5]R. Dhawan, S. Kumari, R. Kumar, S.K. Dhawan, S.R. Dhakate, Mesocarbon microsphere composites with Fe_3O_4 nanoparticles for outstanding electromagnetic interference shielding effectiveness. RSC Adv. **5,** 43279−43289 (2015). https://doi.org/10.1039/C5RA03332B

[S6]S. Teotia, B.P. Singh, I. Elizabeth, V.N. Singh, R. Ravikumar, A.P. Singh et al., Multifunctional, robust, light-weight, free-standing MWCNT/phenolic composite paper as anodes for lithium ion batteries and EMI shielding material. RSC Adv. **4,** 33168−33174 (2014). https://doi.org/10.1039/C4RA04183F

[S7]H. Wang, K. Zheng, X. Zhang, X. Ding, Z.X. Zhang et al., 3D network porous polymeric composites with outstanding electromagnetic interference shielding. Compos. Sci. Technol. **125,** 22−29 (2016). https://doi.org/10.1016/j.compscitech.2016.01.007

[S8]D.X. Yan, H. Pang, B. Li, R. Vajtai, L. Xu et al., Structured reduced graphene oxide/polymer composites for ultra-efficient electromagnetic interference shielding. Adv. Funct. Mater. **25,** 559−566 (2015). https://doi.org/10.1002/adfm.201970288

[S9]N. Agnihotri, K. Chakrabarti, A. De, Highly efficient electromagnetic interference shielding using graphite nanoplatelet/poly(3,4-ethylenedioxythiophene) -poly(styrenesulfonate) composites with enhanced thermal conductivity. RSC Adv. **5,** 43765−43771 (2015). https://doi.org/10.1039/C4RA15674A

[S10] S. Pande, A. Chaudhary, D. Patel, B.P. Singh, R.B. Mathur, Mechanical and electrical properties of multiwall carbon nanotube/polycarbonate composites for electrostatic discharge and electromagnetic interference shielding applications. RSC Adv. **4,** 13839−13849 (2014). https://doi.org/10.1039/C3RA47387B

[S11] M.H. Al-Saleh, W.H. Saadeh, U. Sundararaj, EMI shielding effectiveness of carbon based nanostructured polymeric materials: A comparative study. Carbon **60,** 146−156 (2013). https://doi.org/10.1016/j.carbon.2013.04.008

[S12] M. Arjmand, T. Apperley, M. Okoniewski, U. Sundararaj, Comparative study of electromagnetic interference shielding properties of injection molded versus compression molded multi-walled carbon nanotube/polystyrene composites. Carbon **50,** 5126−5134 (2012). https://doi.org/10.1016/j.carbon.2012.06.053
